# Burden of Disease in Refugee Patients with Diabetes on the Island of Lesvos—The Experience of a Frontline General Hospital

**DOI:** 10.3390/ijerph21070828

**Published:** 2024-06-25

**Authors:** Nikolaos Bountouvis, Eirini Koumpa, Niki Skoutarioti, Dimitrios Kladitis, Aristomenis K. Exadaktylos, Charalampos Anitsakis

**Affiliations:** 1 Department of Internal Medicine, Diabetes Outpatient Clinics, General Hospital of Mytilene “Vostanio”, 83100 Mytilene, Lesvos, Greece; 2 Department of Emergency Medicine (Research), Inselspital, Bern University Hospital, University of Bern, 3010 Bern, Switzerland; aristomenis.exadaktylos@insel.ch; 3 Department of Diabetes, Endocrinology, Nutritional Medicine and Metabolism, Inselspital, Bern University Hospital, University of Bern, 3010 Bern, Switzerland

**Keywords:** diabetes, refugees, Lesvos refugee camps, barriers to accessing healthcare

## Abstract

Diabetes mellitus is a non-communicable disease which poses a great burden on refugee populations, who are confronted with limited access to healthcare services and disruption of pre-existing pharmacological treatment. Aims: We sought to evaluate the degree of hyperglycaemia in refugees with known or recently diagnosed diabetes, to assess cardiovascular comorbidities and diabetes complications, to review and provide available therapeutic options, and to compare, if possible, the situation in Lesvos with other locations hosting refugee populations, thus raising our awareness towards barriers to accessing healthcare and managing diabetes in these vulnerable populations and to propose follow-up strategies. Methods: We retrospectively studied 69 refugee patients (68% of Afghan origin, 64% female) with diabetes mellitus (81% with type 2 diabetes), who were referred to the diabetes outpatient clinics of the General Hospital of Mytilene, Lesvos, Greece, between June 2019 and December 2020. Age, Body Mass Index, diabetes duration, glycaemic control (HbA1c and random glucose), blood pressure, estimated renal function, lipid profile, diabetes complications and current medication were documented at presentation and during subsequent visits. Results: For all patients with type 1 diabetes and type 2 diabetes, age at presentation was 17.7 and 48.1 years, BMI 19.6 kg/m^2^ and 28.9 kg/m^2^ and HbA1c 9.6% and 8.7%, respectively (all medians). One-third (29%) of patients with type 2 diabetes presented either with interrupted or with no previous pharmacological treatment. Insulin was administered to only 21% of refugees with poorly controlled type 2 diabetes. Only half of the patients (48%) with hypertension were taking antihypertensive medication and one-sixth (17%) were taking lipid-lowering medication. Forty-two per cent (42%) of patients were lost to follow-up. Conclusions: Our results showed that a significant portion of refugees with diabetes have either no treatment at all or have had their treatment discontinued, that insulin is still underutilised and that a significant portion of patients are lost to follow-up. It is essential to enhance our ability to identify refugees who may be at risk of developing diabetes or experiencing complications related to the disease. Additionally, it is important to expand access to crucial treatment and monitoring services. By improving our policies for managing non-communicable diseases, we can better support the health and well-being of these vulnerable populations. Furthermore, it is vital to recognize that Greece cannot bear the burden of the refugee crisis alone; international support and collaboration are necessary to address these challenges effectively.

## 1. Introduction

As more than 100 million people [1] from around the globe have been forcibly displaced because of fear of persecution, conflict, violence or violation of human rights, there has been growing concern about the impact of non-communicable diseases (NCD) among refugee populations, particularly cardiovascular diseases (CVD) and diabetes [2].

On the one hand, diabetes and other NCDs are more prevalent in refugees than in non-refugee immigrants or host populations [3,4]. In addition, any pre-existing treatment may be interrupted, especially for refugees temporarily hosted in camps, who form the core of our study population, as they have limited access to medication and healthcare services in areas where they arrive. These factors may aggravate the disease load and trigger acute and chronic complications.

Moreover, healthcare workers at the refugee camps are often comprised of non-governmental organizations (NGOs) and volunteers—and the local healthcare structures (hospitals, health centres) may focus on infectious diseases [5], dental or other urgent cases. As a consequence, many chronic diseases may be poorly addressed, and left undiagnosed or untreated. This may greatly increase the health burden on these populations and on the local healthcare system [6].

Last, but not least, strategies for monitoring and follow-up may be insufficient, as the refugees are continuously being relocated and the health systems of the host countries may be inadequately prepared or coordinated and rely on lengthy administrative procedures. Thus, management of chronic diseases, such as diabetes mellitus, may be suboptimal.

### 1.1. Lesvos as a Path for Refugees

**Figure 1:**
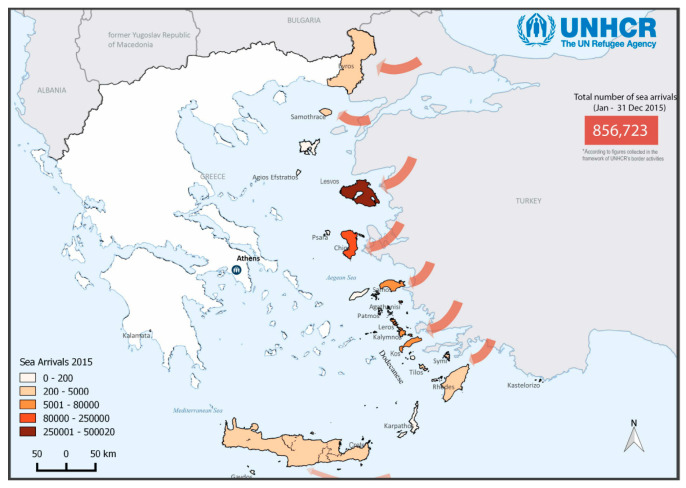
Sea arrivals in Greece January–December 2015 (source: UNHCR Operational Data Portal).

The Greek island of Lesvos (with approximately 84,000 inhabitants in 2021) is located in the northeastern Aegean Sea and is partially surrounded by the shores of Turkey, which in 2022 hosted the largest refugee population worldwide (3.6 million) [1]. Lesvos has in the past 10 years become one of the main entrance points for hundreds of thousands of asylum seekers (approximately 500,000 total sea arrivals in 2015; Figure 1—current and past data retrieved from UNHCR’s Operational Data Portal (ODP); “https://data.unhcr.org/en/situations/mediterranean/location/5179 (accessed on 23 January 2024)”, who mainly originate from Afghanistan (45% of the sea arrivals on Lesvos in 2023), as well as Syria, Eritrea, Somalia and Palestine. They attempt to reach Europe in overcrowded, inflated boats, but end up in refugee camps, formerly known as Reception and Identification Centres (RIC) and today as Closed Controlled Access Centres (CCAC), where they remain until their refugee status is determined and sometimes for several months afterwards.

**Figure 2:**
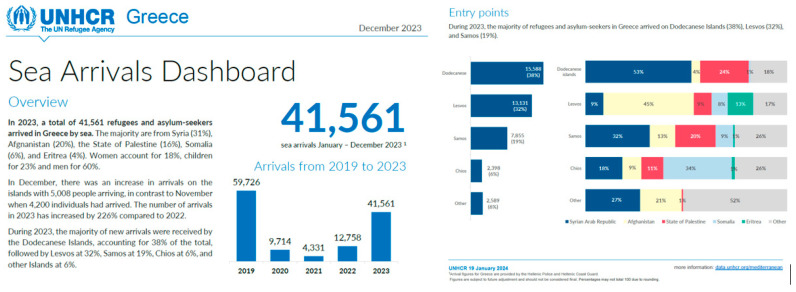
Sea arrivals between 2019 and 2023 (source: UNHCR Operational Data Portal).

After a considerable 2-year drop in the number of new entrances during the COVID-19 pandemic (March 2020–April 2022), there has been an upwards trend since summer 2023. Approximately 13,000 sea arrivals on Lesvos were documented in 2023, a more than 2-fold increase compared to 2022, and this is expected to rise further due to the ongoing humanitarian crisis in Gaza (Figure 2).

### 1.2. Objective

The aims of our study were to estimate the degree of hyperglycaemia in refugees and asylum seekers with known or recently diagnosed diabetes, to assess co-existing cardiovascular risk factors and the presence of diabetes complications, to review the current treatment, if any, to provide the best available therapeutic options and to propose possible follow-up strategies. Moreover, we attempted, where possible, to compare the situation in Lesvos with other locations hosting refugee populations, in order to provide a broader understanding of the challenges and potential solutions in accessing healthcare and managing chronic conditions like diabetes due to displacement, inadequate healthcare systems and the transient nature of refugee status.

## 2. Materials and Methods

We retrospectively studied asylum seekers with diabetes mellitus, who were referred from the RICs of Moria and/or Kara Tepe or from locally attached NGO-healthcare facilities to the emergency department or the diabetes outpatient clinics of the General Hospital of Mytilene “Vostanio”, the leading regional hospital on Lesvos, between June 2019 and December 2020.

The diabetes outpatient clinics team, which is part of the Department of Internal Medicine, comprises medical practitioners specialised in endocrinology, diabetes and internal medicine, as well as a registered nurse with long experience in the management of patients with diabetes mellitus. A registered nutritionist also supports the team.

The study was approved by the Local Ethics Committee (Scientific Board) of the General Hospital of Mytilene. Informed consent was waived. Data were retrospectively retrieved from patient records of the hospital’s diabetes outpatient clinics. All patients who were referred to our diabetes outpatient clinics were included in the study. Women with gestational diabetes or pregnant women with pre-existing type 2 diabetes were excluded from the study.

Glycaemic control was assessed with HbA1c and/or random capillary and/or serum glucose measurements, both at presentation and during subsequent visits. HbA1c Point of Care (POC) testing was not performed. Body mass index (BMI), arterial blood pressure, estimated glomerular filtration rate (eGFR), lipid profile, capillary blood ketone testing, urine albumin–creatinine ratio (UACR), foot examination (monofilament and vibration polyneuropathy testing) as well as retinal examination (performed by ophthalmologists at the ophthalmology outpatient clinics of the hospital) were documented.

This study was not intended to document statistical significance or to perform within-subjects comparisons, but was based on real-life data and every effort was made to improve the glycaemic control and the clinical status of the patients. Continuous variables are presented as medians (range) and categorical variables as frequencies. The descriptive analysis was performed with the JASP^®^ open-source statistical software (jasp-stats.org).

## 3. Results

Sixty-nine, 44 (64%) female and 25 (36%) male patients were included in the study. Fifty-six patients (81%) presented or were diagnosed with type 2 diabetes and 13 (19%) with type 1 diabetes. Approximately two-thirds (*n* = 47, 68%) were of Afghan origin (Table 1).

For all patients with type 1 diabetes and type 2 diabetes, age at presentation was 17.7 and 48.1 years, BMI 19.6 kg/m^2^ and 28.9 kg/m^2^, HbA1c 9.6% and 8.7% and random capillary or serum glucose 386 mg/dL and 271 mg/dL, respectively (all medians). Median age and BMI for female patients with type 2 diabetes were 49.6 years (range 17.4–70.7 years) and 30.2 kg/m^2^ (range 24.0–44.5 kg/m^2^), respectively; 90% of patients had an eGFR above 60 mL/min (median 106.5 mL/min).

About half of the patients with documented blood pressure measurements at the first visit (53% 23/43) were considered to have arterial hypertension (>140/90 mmHg). Most of these patients had type 2 diabetes. Approximately 48% of these patients (11/23) were already taking antihypertensive medication and only one-sixth (17%) were taking lipid-lowering medication.

About a quarter (3 out of 13, 23%) of patients with type 1 diabetes came to the emergency department with diabetic ketoacidosis, and 7% (4/56) of patients with type 2 diabetes needed inpatient treatment due to a hyperglycaemic hyperosmolar state.

Two patients with type 2 diabetes and one patient with type 1 diabetes had signs of diabetic retinopathy and four patients had clinical features of diabetic polyneuropathy. Two patients had foot ulcers, four patients with type 2 diabetes screened positive for albuminuria and one subject underwent dialysis. Where percentages are not reported, it is because of missing data, which would render any descriptive statistical parameter rather inaccurate. Tobacco use was not documented.

Sixteen of 56 (29%) patients with type 2 diabetes presented either with interrupted or no previous antidiabetic medical treatment. A total of 82.5% of the pharmacologically treated patients (*n* = 33) had at least one oral antidiabetic medication, either metformin and/or sulphonylureas, and 30% (*n* = 12) were under insulin treatment, mainly premixed regular with NPH insulin.

Forty-two per cent (42%) of patients (*n* = 29) had no subsequent visits and were lost to follow-up. Patients who presented at at least one subsequent visit were followed up for a median of 9.5 weeks. Median HbA1c at follow-up was 7.2% for patients with type 2 diabetes and random glucose was 229 mg/dL and 193 mg/dL for patients with type 1 diabetes and type 2 diabetes, respectively.

The number of patients with type 2 diabetes, to whom insulin was administered, considerably increased during follow-up (70% vs. 21% at presentation, Table 2). It was also possible to administer relatively newer antidiabetic medications, such as GLP-1 analogues (6/33, 18%), SGLT-2 inhibitors (4/33, 12%) and DPP-4 inhibitors (6/33, 18%), usually in combination with metformin (23/33). Documented statin use increased among refugee patients with type 2 diabetes seen at least once after the first visit, though not significantly in absolute numbers (21%, 7/33).

## 4. Discussion

### 4.1. Prevalence of Diabetes and Severity of Hyperglycaemia

Diabetes mellitus affects more than 500 million people worldwide, with a higher age-standardised rate in the Middle East and North Africa (9.3%). It is estimated that it will affect more than 1.3 billion people by 2050, with a projected prevalence of >16% in these regions of interest, where a significant proportion of asylum seekers originate [7].

Doctors of the World (MdM) reported that the prevalence of diabetes among refugees in camps on mainland Greece was less than 2% [8]. We did not examine the prevalence of diabetes in Lesvos’ refugee camps nor did we compare the magnitude of hyperglycaemia between refugees of different origins, although there may be differences in the burden of NCDs for refugees from different countries.

However, a higher prevalence of self-reported diabetes has been documented in humanitarian entrants compared with other permanent migrants or the host population in different countries [3,9,10]. This can be particularly true for female patients over the age of 40 [2,4,11]. Although it is estimated that approximately 60% of the refugee population in the Greek Aegean islands is currently male, the predominance of female patients examined in our diabetes outpatient clinics may reflect the documented trend towards a higher prevalence of diabetes among the female refugee population, apart from a random and sampling bias [12]. Gender differences in the prevalence of diabetes have been particularly observed in the WHO European Region [13] and may suggest that gender-oriented actions are necessary to prevent the development of diabetes and promote its early detection among the refugee population.

We suggest that screening for diabetes should take place in all refugees above 35 years of age and in younger refugees at particularly high risk for developing diabetes (history of gestational diabetes in women or BMI over 25 kg/m^2^) [14] (Table 3).

### 4.2. Comorbidities and Diabetes-Related Complications

Half of our refugee patients with diabetes were diagnosed with arterial hypertension, but only half of these already received antihypertensive treatment and even fewer lipid-lowering medications. This was in contrast to other studies [15], in which the majority (>80%) of patients with diabetes and hypertension, albeit at older ages, were currently treated with at least one medication for hypertension and dyslipidaemia, and reflects the poor detection of cardiovascular comorbidities in camp-dwelling refugees with diabetes.

Few patients presented with acute complications of diabetes, mainly diabetic ketoacidosis in refugees with type 1 diabetes and severe hyperglycaemic crises in refugees with type 2 diabetes. Admissions to the Department of Internal Medicine aimed to resume or implement insulin therapy in a safe, albeit non-optimal, hospital environment.

Screening for chronic complications such as diabetic retinopathy, nephropathy (including albuminuria screening) and neuropathy can be performed either at the first visit or, if not urgently indicated, at a subsequent visit. The low absolute number of refugees screened and/or diagnosed with chronic complications in our setting may be due to the high proportion of patients lost to follow-up, whose scheduled screening never took place. However, the expected proportion of patients with at least one microvascular complication could be as high as 20% [16].

It should be noted that it is still difficult to estimate the exact duration of diabetes in refugee populations and, in particular, the precise duration of uncontrolled diabetes. It is generally recognised that there is an association between delayed treatment and control of diabetes and an increased risk of long-term complications, the so-called “bad glycaemic legacy”. We hypothesise, hopefully correctly, that the patients we saw at our clinics had been chronically receiving suboptimal treatment, for various reasons (delayed diagnosis, displacement, poor medication availability, underestimation of elapsed time since the diagnosis of diabetes) [17,18].

We suggest that refugee patients who have diabetes (or another NCD) of any duration or control at the time of presentation should be considered to have long-standing disease, should be stratified as being at high or very high risk for developing microvascular and macrovascular complications, and should be screened accordingly and treated aggressively for coexisting cardiovascular risk factors, such as arterial hypertension and dyslipidaemia.

### 4.3. Diabetes Medication, Insulin Use and Adherence to Treatment

The interruption of previously established antidiabetic medication, especially insulin, is one of the most important factors leading to the deterioration of diabetes. In patients with type 1 diabetes, insulin treatment should be prioritised, as any discontinuation in its supply can lead to life-threatening diabetic ketoacidosis. Glycaemic control crucially depends on the timely identification of patients with type 1 diabetes and universal access to insulin for all patients, including those with poorly controlled type 2 diabetes [19].

Most refugee patients with type 2 diabetes attending our outpatient clinics had previously been treated with metformin and sulphonylureas (mainly slow-release gliclazide). This reflects the lower cost and higher penetrance of these drugs in the treatment of diabetes in non-specialised settings, which has also been reported in similar studies [20]. However, it could also be related to the limited availability of other suitable or essential [21] drugs, such as SGLT2-inhibitors (i.e., empagliflozin) or incretin-based treatments (DPP-4 inhibitors and, to a lesser extent, GLP-1 receptor analogues, as they are much more expensive). Lack of experience in prescribing and educating patients in the use of insulin and/or newer antidiabetic treatments, as well as underestimating the ability of patients to self-administer insulin or other injectable medications, can further delay the implementation of the most appropriate pharmacological treatment [22].

As regards the use of newer drugs, SGLT2 inhibitors have been shown to be potentially cost-effective in reducing diabetes complications in low- and middle-income countries [23], both in patients with established cardiovascular or renal disease and in newly diagnosed patients with type 2 diabetes without CVD [24]. Therefore, early implementation of SGLT-2 inhibitors should also be seriously considered in the context of a humanitarian crisis.

Some patients in our study exhibited inadequate glycaemic control even though they were already being treated with insulin (mostly premixed human insulin). Their glycaemic control could be significantly improved by immediately intensifying treatment and switching to a basal-bolus regimen with long-acting and preprandial insulin, as well as early initiation of insulin treatment for insulin-naïve patients, including insulin biosimilars.

Biosimilar insulin analogues have also been included in the WHO list of Essential Medicines [21]. In addition, it has been shown that various formulations of human insulin and insulin analogues can be stored at fluctuating temperatures between 25 and 37 °C and still maintain their effect during the usual four-week period of use [25]. These temperatures, which are not uncommon on the Greek islands in summer and early autumn, should not be regarded as an obstacle to the timely initiation of insulin administration.

Insulin is however still marketed by a few companies and its price remains disproportionately high, given the global burden of disease. Efforts should aim at improving the affordability and accessibility of insulin for a broader range of patients [26].

It is worth noting that all of our patients receiving insulin therapy were treated with insulin pens. Patients who were injecting insulin in ampoules were switched to insulin pens, thus providing a more personalised approach, greater patient comfort and autonomy, as well as ensuring some basic standards of hygiene. However, this practice is not always feasible, as access to insulin pens is associated with higher costs. Mytilene General Hospital provided insulin pen needles at all times to all patients attending our diabetes outpatient clinics.

Although not documented in our study, compliance with treatment was satisfactory, particularly in patients treated with insulin, as was also shown in a study by Médecins Sans Frontières (MSF) in a refugee camp in Lebanon, in which around 70% of patients receiving insulin showed moderate-to-high treatment compliance [27]. Compliance is a very important factor in the success of any medical intervention and its optimisation should be given special consideration by all healthcare providers at the first and all subsequent visits.

### 4.4. Self-Monitoring of Blood Glucose (SMBG) and Diabetes Technology

The General Hospital of Mytilene offered all patients self-monitoring blood glucose (SMBG) equipment (glucose meters, lancing devices, lancets and up to 200 glucose-test strips/month, depending on the type of treatment and monitoring frequency required). Some patients were already wearing SMBG devices provided by NGOs. A 10-year-old Afghan patient with type 1 diabetes was offered continuous glucose monitoring (CGM) for a limited period of time—due to cost considerations.

Self-monitoring of blood glucose (SMBG) is universally recognised as one of the most important components of insulin therapy. The provision of diabetes diagnostics (capillary glucose and HbA1c testing) remains suboptimal in the setting of humanitarian crises [28]. Every patient with insulin-treated diabetes, especially children and young patients with type 1 diabetes, should be provided with appropriate SMBG equipment. It has been shown that personal SMBG testing devices improve disease management and reduce negative outcomes and this was why they were included in the WHO Essential Diagnostics List [29].

According to the American Diabetes Association Standards of Care, the initiation of continuous glucose monitoring (CGM) should also be offered to people with type 1 diabetes early in the disease, even at the time of diagnosis [30]. The implementation of CGM in Syrian refugee children and adolescents (under 16 years old) with type 1 diabetes hosted in Lebanon, as reported by the MSF, was shown to be feasible in a humanitarian setting and was associated with rarer hypoglycaemic events [31]. One recent study was performed on child refugees in Czechia from the Ukraine war with type 1 diabetes. This found that CGM was associated with a substantial improvement in glycaemic control [32].

Continuous subcutaneous insulin infusion (CSII) and automated insulin delivery (AID) systems are also known as insulin pumps or (hybrid) closed-loop systems and have been reportedly underutilised in refugee children with type 1 diabetes compared with native paediatric patients in Germany and Austria [33]. In less developed countries, such as Jordan, insulin pumps or CGM were, until recently, not reimbursed [34].

The case of Eritrean stateless children in Israel showed [35] that CGM can be successfully implemented and lead to positive outcomes regardless of their temporary or permanent legal/asylum status or the perceived “differences” in routine medical care [36], with which medical professionals may feel confronted.

As the use of CGM and/or AID systems is likely to increase in the future, health policymakers and reimbursement bodies should encourage research to develop reliable and widely affordable devices, conduct cost-effectiveness analyses tailored to the current and projected global disease burden, and establish more uniform reimbursement criteria and guidelines that will allow diabetes technology to be made available to a broader range of beneficiaries [37].

In our opinion, refugee patients, especially children with type 1 diabetes, should receive the same level of care and the same treatment options as native patients. Moreover, their ability to manage diabetes technology should be considered a priori at least equal to that of native patients; otherwise, the introduction of newer and probably more effective treatment options could be inappropriately hindered.

### 4.5. Nutrition and Physical Activity

In refugees in camps and neighbouring structures, a diet rich in sodium, carbohydrates (cereals and derivatives) and sugars, but lacking in fresh vegetables and fruits, and provided only a few times daily, may both create a state of food insecurity, with reduced micronutrient and protein intake, and simultaneously contribute to increased insulin resistance [38]. This may predispose them to the development or exacerbation of pre-existing diabetes and hypertension.

Furthermore, poor hygiene standards and the reported poor quality of the meals provided, but more importantly, the inability to prepare, cook and consume their meals as they wish, could also negatively impact the way food evokes feelings, memories and emotions at both individual and social levels among asylum seekers [39], and with unknown, albeit suspected, consequences for their physical and mental health.

In our study, access to food for asylum seekers in camps was limited to meals provided by catering services in the camp, food supplied by NGOs outside the camp, and out-of-pocket purchases from supermarkets located between 10 min and an hour’s walk from the Kara Tepe and Moria camps, respectively. To our knowledge, no cash transfers were offered to asylum seekers with diabetes or other NCDs on Lesvos, apart from what they received (EUR 90/month/person) while their legal status was being assessed. Moreover, it has not been proven that cash transfers alone are sufficient to improve the health outcomes of patients with diabetes in a humanitarian setting [40].

Most of our patients with type 2 diabetes were overweight or obese. This could reflect an ethnic predisposition to adiposity, as well as the sedentary lifestyle and decreased physical activity, which could have probably been aggravated by residing in camps [41], post-traumatic stress or by cultural and socioeconomic barriers related to the challenging adjustment to unfamiliar urban environments [42].

Unsatisfactory nutritional and health status was also observed in refugees after (re)settlement in host countries [43]. Socioeconomic and linguistic factors [44], lack of infrastructure for food storage and preparation, and cultural and family traditions were cited as the main barriers to healthy eating among refugees in urban areas [45]. Although resettled refugees recognise the importance of physical activity, there are numerous barriers to wider uptake, such as lack of time, job insecurity, unfavourable living conditions and psychological stress [46].

Lifestyle interventions should be implemented to prevent weight gain in this population. Such interventions must be multi-level and may help refugee patients overcome structural and behavioural barriers to healthy eating and improve their feeling of social integration as well as their confidence in managing diabetes [47].

It has been shown that strategies promoting physical activity had a positive impact on the mental [48] as well as physical health [49] of participants in the setting of two refugee camps in Greece and in a community-based exercise program in Sweden [50]. Nevertheless, designing these programs and engaging refugee populations can be challenging [51] and has to take into account their cultural heterogeneity and their individual needs.

### 4.6. Patients Lost to Follow-Up and Barriers to Accessing Healthcare

Most of our patients had already been diagnosed with diabetes before entering Greece. The management of NCDs in the context of a humanitarian crisis can be affected by a plethora of barriers to disease control that arise from an increased risk of diabetes due to ethnicity or educational status, lower health literacy, limited access to healthcare services due to costs or legal status [52] and extend up to the inert or underprepared health systems of the host countries [53].

A large proportion of patients in our study (42%) were lost to follow-up. This high rate has also been observed in other studies and may range from 16% among Syrian refugees in Lebanon [12] to 49.2% in a Jordanian University Hospital [54] and can sometimes exceed 50%, as reported among Ukrainian refugees with type 1 diabetes [32].

Losses to follow-up were assumed to be associated with the continuous displacement or the relatively early transfer of asylum seekers with chronic diseases to the mainland, in order to benefit from better housing conditions and availability of health services. However, other, lesser-known causes may also be associated with loss of contact with patients, emphasising the importance of appropriate monitoring strategies to ensure the continuity of their care [55].

### 4.7. Language Barriers and Interpreting Services

Linguistic differences act as a well-recognised barrier to accessing healthcare services. All patients of our study attended the hospital’s diabetes outpatient clinics in the presence of certified interpreters, whose work was crucial for transmitting important medical information to a vulnerable population and for maintaining confidentiality during medical consultations.

Certified interpreters, exclusively provided by NGOs, without—to our knowledge—having had any relevant formal training, contributed to the initiation and titration of insulin, showing that insulin treatment should not be withheld because of perceived language barriers, as reported in a refugee setting in Malaysia [20].

The role of interpreters in educating patients in self-monitoring techniques (SMBG) proved to be equally important and helped to accelerate treatment implementation, in contrast to other reports, where treatment delays were partially attributed, to the interpreters’ poor knowledge of diabetes [56].

Access to interpreting services, both directly or remotely (telephone or video conference), should be strongly encouraged. Remote interpreting is promising in reducing costs as well as in providing less exposure for the patient and less emotional involvement for the interpreter, although it is still underutilised [57]. Interpreters should, however, be appropriately trained in working with potentially traumatised patients, because the use of language assistance services can prove to be an additional stressor for this vulnerable population [58].

### 4.8. Distance, Location and Transportation Barriers

Distance and transportation have also been reported as factors that influence both the choice of healthcare providers as well as the regularity of medical consultations [59]. While the current refugee camp of Kara Tepe is located approximately 5 km from Mytilene town centre (Moria was located a bit farther), a recently designed Closed Control Access Centre (CCAC) in the Vastria region of Lesvos, located more than 30 km from the town centre, could further limit access to healthcare services, with detrimental consequences to the physical as well as mental health of refugees and asylum seekers.

Migration and asylum authorities should carefully examine location-related barriers to accessing healthcare and ensure that asylum seekers with NCDs are not isolated or excluded from receiving proper care.

### 4.9. Social Security Number

It is worth mentioning that almost all asylum seekers to whom insulin analogues or newer antidiabetic medicines could be administered possessed a temporary Social Security Number or Foreigner’s Temporary Insurance and Health Coverage Number (PAAYPA), which made official electronic prescriptions possible. Asylum seekers who were not provided with a temporary Social Security Number relied almost exclusively on third-party cost coverage, which rendered the implementation of insulin or newer medication more difficult—due to its substantially higher cost.

We strongly believe that a regional or European (in the case of EU Member States) Social Security Number should accompany all asylum seekers throughout their presence in host countries, regardless of the acceptance or rejection of their asylum application. This could enable healthcare professionals to provide appropriate support and monitoring as well as the authorities to design more effective health policies.

Legislation on healthcare for asylum seekers should also explicitly include NCDs, such as diabetes, and no room for confusion should be left, with respect to the circumstances (acute or chronic) in which patients with diabetes can receive medical or pharmaceutical treatment. These patient populations are at increased risk of adverse health outcomes, while return to their country of origin is unlikely, as long as conflict situations or other causes that led to their initial displacement persist.

### 4.10. The Role of Community Health Workers (CHW) and NGOs

For Palestinian refugees in West Bank camps [60] as well as for Syrian refugees in Jordan [11], it has been shown that if community health workers (CHW) and volunteers (CHV) are involved in the management of NCDs, such as hypertension and diabetes, this can benefit disease control parameters, medication availability and adherence, referrals for chronic or acute complications and psychosocial support. This applies to refugees living both inside or outside of camps.

Refugee outreach volunteers (ROVs) are a community-based health workforce for refugee communities endorsed by UNHCR, who must demonstrate their wish to help their community, and can be screened for literacy and interest and then trained on NCDs, thereby developing health reference points in the settlements. ROVs can perform screening and CVD risk stratification, record clinical data in mobile applications and refer refugee patients with higher cardiovascular risk to primary healthcare facilities, as shown among Syrian refugees in Lebanon [61].

NGOs and charitable organisations are often regarded as trustworthy providers of direct care and information. They can play an important role in strengthening advocacy in the context of healthcare provision to refugees, in addressing socio-economic disparities and also in increasing compliance with medical advice, as has also been demonstrated among undocumented migrants with diabetes [62].

Multidisciplinary care models that integrate coordination of clinical and community health work (I-Care) have been shown to improve longer-term health equity for refugee patients with diabetes, increase compliance with treatment and screening for diabetes complications, and build trust and confidence in culturally and linguistically diverse patient populations [63].

In our study, NGO-appointed CHW contributed by ensuring appropriate treatment and this could therefore be provided to refugees with or without a Social Security Number and, thus, no access to prescription medications. They could help by arranging transportation, interpreters and costs related to specialist consultations, either in public (free) or private healthcare settings.

Access to healthcare volunteers and CHW in the refugee camps was, however, limited during the quarantine measures of the COVID-19 pandemic, a phenomenon, which could have impeded the communication between healthcare providers and the local authorities and rather obstructed the patients’ follow-up.

### 4.11. Healthcare Provider Availability and Refugee-Oriented Consultations

Free medical consultations in refugee camps as well as in urban settings can be of vital importance because their objective is to meet the medical needs of vulnerable populations, who would otherwise have little or no access to health care.

In the setting of the refugee camps of Moria and Kara Tepe, as well as in the PIKPA camp, the contribution of on-site medical volunteers or practitioners recruited by NGOs and the National Public Health Organisation was of vital importance to the early diagnosis and treatment of patients with diabetes and other NCDs.

However, there may be too few medical practitioners, perhaps because recruitment is difficult as the refugees may be in remote areas. What is more, the medical staff may lack specific training in treating patients with diabetes, especially in the context of a humanitarian crisis. Thus, there may be delays in referrals. Praksis, an NGO focusing on medical humanitarian action and social support, has found that rates of diagnosing and specialist referrals may be lowered on the Greek Islands, showing that both location and availability play an important role in providing healthcare to refugees [64].

Incomplete information about access to healthcare services and treatment costs may also impair the continuity of treatment [65]. Moreover, refugees’ access to healthcare may be hampered by delays in the response of the local health authorities and possibly also by the availability of appointments for asylum seekers in local healthcare facilities. This has contributed to the establishment of parallel medical services from NGOs, which should be supported and strengthened by local governments and international organisations, in order to promote coordination in addressing refugees’ health needs.

### 4.12. Cultural Competency and Training of Healthcare Professionals

Healthcare professionals in the host countries usually lack adequate training in refugee and migrant health, which poses special challenges for those involved. Healthcare providers should be culturally competent and sensitive to the unique needs faced by refugees with NCDs. This includes not only addressing language barriers but also respecting cultural beliefs and practices, and providing personalised care that takes into account refugees’ backgrounds and experiences. This suggests that innovative recruitment methods may be necessary in coping with linguistic and cultural barriers to accessing healthcare—such as recruiting specialist staff from outside the host countries or from among the refugee populations [66].

Access to responsive, people-centred health systems is essential to ensure appropriate health care for refugees throughout the process of settlement. There is an increasing global interest in research aimed at promoting refugee and migrant health, in training healthcare professionals, as well as growing awareness among health authorities on how to identify, address and overcome challenges in the provision of healthcare to refugees and migrants.

Some of the systemic shortcomings in supporting refugees in need may be addressed by involving and training medical students in fundamental tasks such as diabetes management (including dietary management, glucose checking, medication management, and complications screening), as well as in other aspects of humanitarian crises, for instance in organising telemedicine platforms, in founding charities or in ensuring that medical supplies are transferred to conflict areas [67].

### 4.13. Monitoring Strategies and mHealth

Short- and long-term monitoring strategies are urgently needed to ensure the continuity of care in refugees with diabetes and other NCDs (Table 4).

Patients with uncontrolled diabetes should be seen on a more regular basis (weekly or biweekly), especially those treated with insulin, in order to ensure that they understand insulin titration and that they avoid hypoglycaemia. Where staff shortages or location barriers may impede appropriate monitoring, the utilisation of mobile devices, such as smart or portable gadgets (mHealth), can improve interaction with nurses and CHW and the implementation of treatment protocols, screening and referrals.

At the same time, patients should receive appropriate education (>4 sessions/year have been recommended) in an effort to improve their understanding of the disease (health literacy) and techniques for diabetes self-management [22]. mHealth can also facilitate patient education and adherence to treatment.

### 4.14. Limitations and Strengths

This study, while providing critical insights, has several limitations that must be acknowledged.

Firstly, the generalisability of the findings is confined largely to the refugee population on the island of Lesvos. Given the unique socio-economic and healthcare contexts of Lesvos, the results may not be directly transferable to other refugee settings without similar environmental and demographic characteristics.

Secondly, a significant proportion of participants were lost to follow-up, which might introduce bias into the findings. This attrition could impact the reliability and validity of study outcomes, as it remains unclear if those lost had different characteristics or health outcomes compared to those who remained.

Additionally, the observational and retrospective design of the study limits the ability to establish causality. Relying on hospital records and self-reported data without the longitudinal, prospective tracking of outcomes may affect the accuracy of reported diabetes prevalence and management outcomes.

Moreover, the study did not adequately explore the health literacy levels or mental health aspects of the refugee population, especially post-traumatic stress disorder, which are important factors that can significantly influence the management of diabetes and other NCDs. The role of health education and its impact on disease management was not fully explored, which is crucial for designing effective health intervention programs tailored to refugee populations.

Hygiene, immunization status, vector-borne or other infectious diseases that could have complicated the management of diabetes or have been exacerbated by poorly controlled diabetes are not covered. Pregnant women with gestational diabetes or pre-existing diabetes were not included in this study because they can harbour unique features that should have been more extensively analysed. Valuable insights regarding the management of diabetes and obstetric complications in this unique patient population are missing. Our sample did also not include any children under 5 years of age and included a rather limited number of elderly patients, in whom morbidity and mortality may be significantly increased.

Finally, there has been a significant time difference between the present and the reference period and there may have been changes in healthcare provision in the above-mentioned environment. To the best of our knowledge, this is the first study specifically addressing diabetes in refugees on an island or “hotspot” in Greece, which may, in our opinion, provide timely information, as the prevalence of diabetes and other NCDs in refugee populations is expected to rise in the coming years due to geopolitical changes, wars, natural disasters and human rights violations.

Despite these limitations, this study is critically important as it highlights the urgent health needs of a highly vulnerable population under significant stress. It underscores the necessity for targeted healthcare interventions and provides foundational data that can inform policy and improve healthcare delivery in similar contexts globally. By addressing these health challenges, the study contributes to a deeper understanding of how to effectively manage chronic conditions in crisis settings, an area of increasing relevance as global displacement continues to rise.

## 5. Conclusions

Early identification of vulnerable refugees and asylum seekers, prompt intervention with access to insulin and other essential diabetes medications, appropriate monitoring and trained healthcare providers must be standard in any humanitarian response and integrated into routine care. The introduction of standardised clinical and operational guidelines and better coordination between health authorities and institutions, while strengthening the role of NGOs, are key elements of a strategy to reduce the burden of disease and improve the health status of this vulnerable population.

## Figures and Tables

**Table 1:** Patient baseline and follow-up characteristics.

	Type 1 Diabetes		Type 2 Diabetes	
Total (*n*)	13		56	
Afghan origin (*n*, %)	10 (77%)		37 (66%)	
Female (*n*, %)	7 (54%)		37 (66%)	
	Median	Range	Median	Range
Age (years)	17.7	6.3–39.9	48.1	17.4–70.7
Body mass index (kg/m^2^)	19.6	15.4–25.1	28.9	17.5–45.6
Reported diabetes duration (years)	2	0–26	5	0–18
Random blood glucose (RBG) at first visit (mg/dL)	386	234–600	271	88–457
HbA1c at first visit (%)	9.6	7.1–10.7	8.7	5.7–14
eGFR at first visit (mL/min/1.73 m^2^)	-	-	106	14–138
Systolic blood pressure at first visit (mmHg)	113	106–153	127	86–146
Random blood glucose (RBG) at last visit (mg/dL)	228.5	56–294	192.5	95–337
**HbA1c at last visit (%)**	7.3 *	7.2–7.4	7.2	5.6–10.5
**Total duration of follow-up (weeks)**	9.9	0.7–19.9	9.14	1–49

* Only two patients with type 1 diabetes had follow-up HbA1c measurements.

**Table 2:** Anti-diabetic medication and statin use in refugee patients with type 2 diabetes.

	First Visit	Last Visit
	*n* (%)	*n* (%)
No medication	16/56 (29%)	4/33 (12%)
Insulin	12/56 (21%)	23/33 (70%)
Metformin and/or gliclazide	33/56 (59%)	23/33 (70%)
SGLT2-inhibitors	-	4/33 (12%)
GLP-1 receptor analogues	-	6/33 (18%)
DPP-4 inhibitors	-	6/33 (18%)
Statins	4/56 (7%)	7/33 (21%)

**Table 3:** Suggestions for diagnosis and monitoring.

Diabetes and/or gestational diabetes (GDM) history during triage at the entry point and past medication overview (+history of CVD)
Random capillary blood glucose testing for refugees with or at risk of diabetes mellitus (>200 mg/dL suggests overt diabetes)
Point of Care (POC) HbA1c testing for refugees with pre-existing or newly diagnosed diabetes
Capillary blood ketone (β-hydroxybutyrate) testing for patients with type 1 diabetes or with symptoms suggestive of a catabolic state or insulinopenia (polyuria, polydipsia, weight loss)
Serum or capillary creatinine testing and estimation of the renal function (eGRF) and POC albuminuria screening, where available.

**Table 4:** Suggestions for treatment and follow-up.

Provide appropriate nutrition.
Consider insulin (basal) earlier for type 2 diabetes patients, especially when A1C > 9%
Consider SGLT-2 inhibitors early after patient presentation
Provide and educate refugee patients on blood glucose self-monitoring (SMBG) and insulin-injection technique
Consider Continuous Glucose Monitoring (CGM) for every patient with type 1 diabetes
Arrange regular follow-up visits
Refer patients for fundoscopy (consider AI screening programs, where ophthalmology availability is limited)
Provide all patients with Social Security Number to enable access to medication and appropriate tracking
Educate healthcare providers on culturally diverse patient populations

## Data Availability

The data presented in this study are available on request from the corresponding author due to privacy and legal reasons.
